# Free recall in autism spectrum disorder: The role of relational and item-specific encoding

**DOI:** 10.1016/j.neuropsychologia.2007.11.011

**Published:** 2008

**Authors:** Sebastian B. Gaigg, John M. Gardiner, Dermot M. Bowler

**Affiliations:** a Department of Psychology, City University, London, UK; b Department of Psychology, University of Sussex, UK

**Keywords:** Autism spectrum disorder, Medial temporal lobe memory system, Hippocampus, Task support hypothesis

## Abstract

Autism spectrum disorders (ASDs) are characterised by a relatively specific pattern of typical and atypical memory functioning. Convergent behavioural and neuroscientific evidence indicates that this pattern of functioning may be the result of specific impairments in hippocampally mediated relational memory processes, whilst brain-mechanisms mediating item-specific memory processes remain intact. In the current paper we draw on a behavioural paradigm developed by Hunt and Seta [Hunt, R. R., & Seta, C. E. (1984). Category size effects in recall—The roles of relational and individual item information. *Journal of Experimental Psychology: Learning, Memory and Cognition*, *10*, 454–464], which not only allowed us to determine whether individuals with ASD did indeed experience selective difficulties in relational processes, but in addition enabled us to gain insights into the severity of this impairment. Our results suggest that whilst individuals with ASD employ relational memory processes atypically, this impairment seems restricted to situations in which such processes need to be deployed spontaneously to facilitate memory. Under situations that provide environmental support for the processing of relational information, individuals with ASD did demonstrate the ability to employ such processes relatively effectively. These findings provide further support for the ‘Task Support Hypothesis’ and suggest that relational memory processes may in principle be functionally intact despite not being triggered by the same environmental situations as in typical development.

## 1 Introduction

Autism spectrum disorders (ASDs) are clinically defined by difficulties in reciprocal social behaviour and communication and the presence of stereotyped patterns of behaviour and restricted interests (ICD-10: World Health Organisation, 1992; DSM IV-TR: American Psychiatric Association, 2000). In addition to this unique combination of symptomatology, the condition is also characterised by a relatively specific combination of typical and atypical functioning within the domain of memory. Since this patterning of memory functioning cannot be accounted for by the varying degree of language or general intellectual disability that often accompanies the core clinical features of ASD, it is thought to reflect a facet of the broader phenotype characterising the disorder. We propose that a cognitive framework that distinguishes between item-specific and relational memory processes may not only provide a suitable explanation for available behavioural evidence, an idea that we test in the present study, but may also prove useful in guiding future neuroscientific work relating to medial temporal lobe (MTL) functioning in ASD.

On the basis of currently available evidence the patterning of memory functioning in ASD may be summarised as follows. Procedures such as recognition, priming and cued recall generally tend to yield typical levels of performance in ASD (Boucher & Lewis, 1989; Bowler, Gardiner, & Grice, 2000a; Bowler, Matthews, & Gardiner, 1997; Gardiner, Bowler, & Grice, 2003; Minshew, Goldstein, Muenz, & Payton, 1992; Minshew, Goldstein, Taylor, & Siegel, 1994; Tager-Flusberg, 1991). By contrast, free recall paradigms generally lead to diminished performance in this population especially when semantic, syntactic or phonological information is available to aid recall (Bowler, Gardiner, Grice, & Saavalainen, 2000b; Hermelin & O’Connor, 1967; Smith, Gardiner, & Bowler, 2007; Tager-Flusberg, 1991; but see López & Leekam, 2003 for contrary evidence). These free recall difficulties parallel findings from the typical aging literature (e.g. Craik & Anderson, 1999; Craik, Morris, Morris, & Loewen, 1990) and led Bowler et al. (1997) to posit a ‘Task Support Hypothesis’ according to which procedures that provide cues to the remembered material at test attenuate the memory difficulties experienced by individuals with ASD. Bowler, Gardiner, and Berthollier (2004) demonstrated that this framework could account for conflicting results regarding source memory capacities in individuals with ASD where previous studies had observed impaired performance on tests of source recall but undiminished performance on tests of source recognition (e.g. Bennetto, Pennington, & Rogers, 1996; Farrant, Blades, & Boucher, 1998).

Although the task support hypothesis can account for the patterning of performance by individuals with ASD across a variety of memory paradigms, the causes for this greater reliance on support for the retrieval of previous experiences remain to date unknown. Earlier attempts to account for the pattern of intact and impaired memory processes in ASD have often invoked encoding as the source of difficulty. The most influential of these accounts is based on the seminal work of Hermelin and O’Connor (1970) who demonstrated that compared to non-ASD children who demonstrate superior recall for semantically and syntactically organised word sequences, children with autism do not tend to draw on such semantic and syntactic features to aid recall. On the basis of this evidence Hermelin and O’Connor (1970) argued that individuals with ASD do not encode stimuli meaningfully. Although several investigations have supported this hypothesis (e.g. Bowler et al., 2000b; Tager-Flusberg, 1991), three strands of more recent evidence indicates that the encoding difficulties seen in ASD may be more subtle than general problems with processing semantic information *per se*. We will briefly consider each of these in turn.

First, individuals with ASD have been found to be subject to semantically induced memory illusions when Roediger and McDermott's (1995) procedure is used. In such paradigms individuals are asked to try to remember a series of words that include the strongest semantic associates of one non-presented target word (e.g. *bed*, *dream*, *night*, etc. for the target word ‘*sleep*’). Bowler et al. (2000b) and Beversdorf, Smith, Crucian, Anderson, and Keillor (2000) showed that individuals with ASD like typical individuals are more likely to falsely remember the semantically related target words than semantically unrelated words. Although the findings by Beversdorf and colleagues suggested that individuals with ASD may be better at discriminating the illusory target words from actually studied items, the finding that individuals with ASD did experience illusory memories shows that they are sensitive to the semantic associations of the studied words at least to some extent.

The second strand of evidence concerns the observation that individuals with ASD exhibit relatively typical levels of performance following deep levels of encoding (Bowler et al., 1997; Gardiner et al., 2003; Mottron, Morasse, & Belleville, 2001; Toichi & Kamio, 2002). Deep levels of encoding generally involve the processing of semantic aspects of material (e.g. thinking about category membership of words), which typically leads to enhanced memory in comparison to shallower levels of encoding that involve the processing of non-semantic features of material (e.g. counting the number of syllables of words) (Craik & Lockhart, 1972). The finding of typical levels of performance following deep levels of encoding in ASD thus again suggests that under some circumstances such individuals encode semantic aspects of stimuli relatively effectively. Interestingly, studies employing levels of processing paradigms have also tended to note superior performance of individuals with ASD following shallow levels of encoding (e.g. Toichi & Kamio, 2002). This pattern has led Mottron et al. (2001) to suggest that rather than being deficient in processing semantic or ‘higher-level’ conceptual information, individuals with ASD may be superior at processing ‘low-level’ perceptual information and that this processing style may interfere with higher-level conceptual processes in some circumstances. We will return to this argument again later.

The third and final strand of evidence regards a recent set of studies from our laboratory. In this set of experiments we asked participants to study a list of words, each of which was accompanied by a semantically related or semantically unrelated context word (e.g. ‘Wood’ in the context of ‘Tree’ vs. ‘Stone’ in the context of ‘Motor’). Whilst individuals with ASD failed to benefit from the semantic relatedness of to-be-remembered words and simultaneously presented context items on a test of free recall, their performance on a test of recognition was enhanced by such semantic relationships to a similar extent as found in typically developed comparison participants (Bowler, Gaigg, & Gardiner, 2008b). Again this finding suggests that individuals with ASD are sensitive to semantic features of stimuli, at least when test procedures support retrieval.

The apparent contradiction between diminished use of semantic relations to aid free recall and relatively typical use of semantic features of stimuli under certain circumstances may be resolved by means of a closer analysis of what each of the paradigms described above requires of the participant. Experiencing an illusory memory on the basis of studying strong associates of a non-studied word implicates the relation between each studied word and the participant's existing knowledge base (e.g. Item A is associated with Concept X, Item B is associated with Concept X, etc.) and does not rely heavily on processing the relations amongst the studied items (Roediger, Watson, McDermott, & Gallo, 2001). Deeper levels of encoding equally do not necessitate relating studied items to one another but rather require enhanced attention to the semantic properties of each studied item. Finally, performance on tests of recognition has been found to rely more heavily on the ability to draw on information specific to individual items, including their semantic properties, rather than relationships among items (e.g. Anderson & Bower, 1972). In contrast, making efficient use of semantic features of stimuli during free recall tasks relies not only on the ability to process the semantic properties of each item but in addition on the ability to make use of these semantic features to establish associations amongst the items (i.e. Item A is associated to Item B because they are both associated with Concept X).

The foregoing analysis leads us to speculate that individuals with ASD may have specific difficulties in using semantic information that emerge as a result of the relationships between items, whilst their capacity to draw on semantic information that is specific to individual items appears to be intact. This distinction between *relational* and *item-specific* processing has been widely applied to account for a variety of memory phenomena (Anderson & Bower, 1972; Hunt & McDaniel, 1993) and specific difficulties in processing relational information would explain why individuals with ASD rely on greater task support during retrieval. Such difficulties would also explain why individuals with ASD experience fewer episodically defined recollective experiences but somewhat more familiarity based experiences on tests of recognition that employ the ‘Remember/Know’ procedure (Bowler et al., 2000a; Bowler, Gardiner, & Gaigg, 2007; see Gardiner, 2001 for further details on the ‘Remember/Know’ procedure). Recollective experiences require that information be encoded and stored in *relation* to spatial and temporal contextual information whilst familiarity based recognition judgments can be mediated on the basis of available item-specific information alone (see Gardiner, 2001; Tulving, 1985, 2002 for further details). Furthermore inefficient use of organisational strategies such as semantic clustering (e.g. Minshew & Goldstein, 2001) or subjective organisation (Bowler, Gaigg, & Gardiner, 2008a) to facilitate memory in ASD also indicate that this population experiences difficulties in using relationships amongst items to organise their retrieval in free recall.

The suggestion that ASD may be characterised by relatively specific difficulties in relational memory processes has recently also emerged on the basis of neuroscientific evidence (Nicolson, DeVito, Vidal, Sui, & Hayashi, 2006). Since the first direct examinations of the brains of individuals with ASD (Bauman & Kemper, 1985), atypicalities in areas associated with memory processes have repeatedly been documented (see Bachevalier, 1994; Kemper & Bauman, 1998; Palmen, van Engeland, Hof, & Schmitz, 2004 for reviews). Although the findings remain somewhat inconsistent, morphological abnormalities of the hippocampus are relatively well documented in ASD (see Nicolson et al., 2006). Areas surrounding the hippocampus, such as perirhinal, entorhinal and parahippocampal areas have less often been the focus of investigation but the observations by Bauman and Kemper (1985) suggest that at least the entorhinal cortex seems to be less affected than the hippocampus in ASD individuals.^1^ Until recently it has been difficult to relate these pathological findings to the memory difficulties experienced by individuals with ASD because the precise role of distinct medial temporal lobe areas in mediating memory processes was only vaguely understood. Accumulating evidence, however, now demonstrates that relational and item-specific processes are mediated by distinct sub-systems of the medial temporal lobe (MTL) memory system. More specifically, the hippocampus has been identified as the site of domain-general relational memory processes where individual features of an episode are integrated and organised (e.g. Eichenbaum, 2004; Holdstock, Mayes, Gong, Roberts, & Kapur, 2005; Squire, 1992). Areas outside the hippocampus, such as perirhinal, entorhinal and parahippocampal areas, on the other hand, seem to mediate more domain-specific item and contextual processes (e.g. see Davachi, 2006; Mayes, Montaldi, & Migo, 2007 for comprehensive reviews). Of particular interest in relation to ASD is the finding that episodically based recognition judgements that involve the recollection of contextual information (and are impaired in ASD) are primarily mediated by hippocampal processes whilst familiarity based recognition judgements (which are intact in ASD) are mediated by perirhinal processes (Brown & Aggleton, 2001; Davachi, Mitchell, & Wagner, 2003; Davachi & Wagner, 2002; Holdstock et al., 2005). This dissociation, together with the wider memory and neuropathological literature in ASD suggests that the item-specific/relational distinction may provide a useful heuristic device to guide further neuroscientific investigations of MTL pathology in ASD. For such an endeavour to be successful, however, it is necessary to test whether this framework provides an adequate explanation for the behavioural manifestations of memory difficulties in ASD.

In the current paper we test the hypothesis that individuals with ASD are characterised by specific behavioural difficulties in relational memory processes whilst item-specific memory processes are spared. The current paradigm is based on a study by Hunt and Seta (1984), who argued that the efficiency of recalling items from a list of categorised words depended on the availability of both item-specific and relational information. Item-specific information, they suggest, is important in order to effectively distinguish amongst items from within a given category whilst relational information is important in order to recall the category *per se*. In order to test this hypothesis, they asked participants to study a list of words that included varying instances of items belonging to different categories (e.g. 2 Items of Fruit, 4 Professions, 8 Countries, 12 Animals, 16 Furniture). Hunt and Seta (1984) argued that because the relational nature of the items from the relatively small categories in such a list is relatively unobvious, effective recall of these categories depends disproportionately on the availability of relational information. By contrast, effective recall of items from the relatively large categories depends disproportionately on the availability of item-specific information because such information facilitates the differentiation of items within these categories. In support of their hypotheses, they showed that participants who encoded words through a relational orienting task (i.e. sorting words into categories) recalled items from the less obvious categories that were represented relatively infrequently in the study list significantly better than participants who encoded the words through an item-specific orienting task (i.e. rating words on pleasantness). In addition, the relational orienting task facilitated the recall of at least one item from each of the categories (particularly the relatively small categories) supporting the view that relational information is important for the recall of the categories *per se*. By contrast, participants who encoded words through the item-specific orienting task exhibited superior recall of items from the categories that were represented relatively frequently in the study list. In short, whilst the encoding of relational information disproportionately benefits the recall of words from relatively small (relative to other categories in the list) and therefore not very obvious categories, the encoding of item-specific information is disproportionately beneficial for the recall of items from relatively large categories.

In the current experiment we asked participants to study two lists of words that, following Hunt and Seta (1984), consisted of varying instances of members from different categories. For the first list, individuals were simply asked to try to remember as many words as possible for an upcoming free recall test. Following this baseline condition, participants studied a second list whilst carrying out either the item-specific or relational encoding tasks employed by Hunt and Seta (1984). On the basis of evidence showing that typical individuals consistently benefit from semantic and categorical relationships to facilitate recall (e.g. Bousfield, 1953; Bower, Clark, Lesgold, & Winzenz, 1969) we predicted that during the baseline condition, the typical group would tend to rely on relational memory processes. In contrast, and on the basis of the evidence outlined above, we hypothesised that ASD individuals would rely more heavily on item-specific memory processes. Since relational information is particularly important for effectively recalling relatively small categories, we therefore predicted that during the baseline condition, the ASD group would exhibit disproportionate recall difficulties for items from relatively small categories whilst their recall of items from relatively large categories would not be as seriously compromised. In relation to performance following the relational orienting task, our prediction is less specific. If relational memory processes in ASD are impaired to such an extent that they cannot be deployed even when environmental support would facilitate such processes, the disproportionate recall impairment for smaller categories would persist. If, on the other hand, the impairment in relational memory processes is restricted to circumstances in which such processes would need to be deployed spontaneously, the task support hypothesis would predict that a relational orienting task would alleviate the recall difficulties in ASD thereby resulting in a relatively typical level of performance across category sizes. Finally, based on the evidence that individuals with ASD employ item-specific memory processes effectively, we predicted no recall impairment of this group following the item-specific orienting task. To the contrary individuals with ASD may outperform typical individuals in this condition because they may have developed superior skills in item-specific processing in order to compensate for their difficulties in relational processes. The finding that individuals with ASD tend to outperform typical individuals following shallow encoding tasks (e.g. Mottron et al., 2001) would be in line with this suggestion.

## 2 Method

### 2.1 Participants

Twenty individuals with autism spectrum disorder (7 female, 13 male) and 20 typical individuals (7 female, 13 male) took part in this experiment. Participants were individually matched on Verbal IQ as measured by the WAIS-R or WAIS-III^UK^ (The Psychological Corporation, 2002) and groups did not differ on Performance IQ, Full scale IQ or age. Ten participants from each group were randomly allocated to each of the two orienting task conditions (described below) with the constraint that IQ scores and age were similarly distributed across the two conditions. Table 1 summarises these data. All individuals with ASD were diagnosed by local health authorities and/or experienced clinicians, and met DSM-IV-TR (American Psychiatric Association, 2000) criteria for Asperger's disorder or Autistic disorder. The Comparison group was recruited via local newspaper advertisements. Brief interviews ensured that no participant had a history of neurological or psychiatric illness. Individuals gave their informed consent to take part in the study and were paid standard University fees for their participation.

All but four individuals with ASD (two from each orienting task condition), who had been prescribed low doses of antidepressant medication, were free of psychotropic medication. Since the exclusion of these participants and their matched typical individuals did not alter the results significantly, all participants were included in the analysis.

### 2.2 Design and materials

On the basis of Hunt and Seta's (1984) first experiment study lists were constructed from a master pool of words that consisted of 16 words from each of 10 categories selected from the Battig and Montague (1969) category norms. The frequency of the words ranged from 1 to 25 and the average category rank of words was 10 (see Battig & Montague, 1969 for details). The categories of Sports, Clothing, Weapons, Countries and Animals served as set A and the categories of Birds, Kitchen Utensils, Parts of the Body, Fruits and Vehicles served as set B. From each set, 5 study lists were constructed consisting of a total of 42 target items and 8 buffer items to counter primacy and recency effects (4 at the beginning and 4 at the end). Within each list relative category size was manipulated by selecting 2, 4, 8, 12 or all 16 items from the 5 categories (e.g. 2 Sports, 4 Clothing, 8 Weapons, 12 Countries and all 16 Animals). Across the 5 lists each category appeared at each category size once. Buffer items were selected from the categories of Professions and Parts of a Building for set A and Earth Formations and Alcoholic Drinks for set B.

Words were printed in bold, arial font (size 36; Microsoft Word for Windows) in the centre of 8.2 cm × 7.6 cm, laminated cards. The 42 cards constituting the target items were ordered pseudo randomly with the constraint that the average lag between items from the same category be as close to 2 as possible (ranging from 0 to 5).^2^ The buffer items in the beginning and end of the target list were also randomised so that no more than two consecutive words were from the same category. The orders of items in the 5 lists from sets A and B were equivalent in terms of the list position of words from the differently sized categories.

### 2.3 Procedure

Unlike Hunt and Seta (1984) the current experiment included a baseline condition during which participants were presented with one of the study lists (in the form of a deck of cards) from either set A or B and simply asked to try to remember as many words as possible. Participants were allowed to go through the cards at their own pace and the total amount of time they required to do so was recorded. Participants were instructed to put each card face down in front of them after they had tried to remember it and to not look at a card again once it was placed on the table. Immediately after the last word, oral free recall was requested.

Following a 5–10-min break, individuals were given the respective deck of cards from the set not used during baseline, and given instructions for either the item-specific or the relational orienting task employed by Hunt and Seta (1984). For the relational orienting task printed category labels were placed on the table and participants were asked to sort the word-cards into their respective categories. In the case of uncertainty participants were asked to guess what category a word belonged to. For the item-specific orienting task, labels representing a 5-point pleasantness rating scale (very pleasant, a little pleasant, neutral, a little unpleasant, very unpleasant) were placed on the table, and individuals were asked to rate each of the words on this scale orally and not sort the cards underneath the labels. Regardless of orienting task individuals were asked to try to remember as many words as possible and following the last word all materials were cleared from the table and oral free recall was again requested.

## 3 Results

A 2 (ASD vs. Comparison) × 2 (Baseline vs. Orienting Tasks condition) mixed ANOVA of the time participants spent looking through the decks of cards revealed a significant (*F*(1, 36) = 7.96, *p* < .01) effect of condition with participants spending an average of 323 s (S.D. = 404) looking through the cards whilst carrying out the orienting tasks compared to 244 s (S.D. = 254) during the baseline condition. Neither the main effect of group nor the interaction between group and condition were significant. An analysis of the time participants spent looking through the cards during the two orienting tasks revealed no main effects or interaction of the factors group (ASD vs. Comparison) and orienting task (Rate vs. Sort). Since the time participants spent looking through the cards correlated highly (*r* > .65) with overall recall levels for both groups in all conditions, encoding time was entered as a covariate in all subsequent analysis of the recall data (Miller & Chapman, 2001).

### 3.1 Baseline condition

**Fig. 1:**
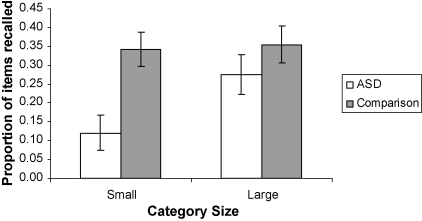
Average proportions of items recalled during the baseline condition from the small 2, 4 and 8 item categories and the large 12 and 16 item categories as a function of group. Error bars show standard errors.

The free recall data for the baseline condition are illustrated in Fig. 1, which gives the average proportion of items recalled from the smaller (i.e. size 2, 4 and 8) and larger (i.e. size 12 and 16) categories for the ASD and Comparison groups. Overall, the ASD group recalled fewer words than the Comparison group (*F*(1, 37) = 8.08, *p* < .01) which was most marked with smaller categories resulting in a significant group by category size interaction (*F*(1, 37) = 6.89, *p* < .05). Thus, in line with our prediction that individuals with ASD would exhibit a recall decrement that would be indicative of specific difficulties in relational memory processes, the ASD group recalled significantly fewer words from the small categories (*t*(38) = 3.37, *p* < .01; Cohen's *d* = 0.96) but not the large categories (*t*(38) = 1.09, ns; Cohen's *d* = 0.30). We note that we have collapsed the recall data into ‘small’ and ‘large’ categories for simplicity and in order to facilitate the calculation of effect sizes. An analysis of the data across the five levels of category size yielded the same significant main effects and interactions (or lack thereof) as those reported above and in the analysis of the data from the orienting tasks below.

As indicated earlier, Hunt and Seta (1984) suggest that the recall of at least one item from any given category (i.e. category availability—CA) represents the availability of relational information during recall, as does the amount of category clustering individuals employ during retrieval. Category clustering, as indexed by the Modified Ratio of Repetition is a simple ratio of the number of category repetitions (i.e. two consecutive items are recalled from the same category) to the total number of items recalled across all categories. As Hunt and Seta (1984) point out, more sophisticated measures of clustering are unsuitable for obtaining measures of organisation for each category size because they are mathematically undefined for a single category. In contrast to these indices of relational information, the number of items participants recall within a particular category (i.e. items per category—IPC) depends on the availability of item-specific information since such information aids the differentiation of individual instances of a particular category. In order to provide further insights into the use of item-specific and relational information to facilitate memory in ASD we computed these measures which are set out in Table 2.^3^

A 5 (category size) by 2 (group) mixed ANCOVA on the number of categories recalled (category availability) revealed a significant main effect for category size (*F*(4, 34) = 15.22, *p* < .001) with larger categories being nearly perfectly recalled whereas the smallest 2-item category was only recalled by 35% of participants. As expected, the ASD group recalled significantly fewer categories (*F*(1, 37) = 10.69, *p* < .01) and this effect was again characterised by a significant interaction between category size and group (*F*(4, 34) = 12.25, *p* < .001). Post hoc nonparametric comparisons showed that the ASD group recalled the small 2 and 4 item categories less often than typical participants (*z* = 2.82, *p* < .01, one-tailed) whilst both groups recalled the larger 12 and 16 item categories nearly perfectly. Although this result needs to be interpreted with caution due to the ceiling performance on larger categories, further evidence for the attenuated use of relational information to facilitate recall in ASD stems from the analysis of the category clustering data. Again this measure increased with category size (*F*(4, 34) = 10.13, *p* < .001) and again individuals with ASD clustered words into their respective categories less than the comparison group (*F*(1, 37) = 5.66, *p* < .05). Again the interaction between group and category size needs to be interpreted with some caution due to the floor performance on smaller categories. However, as the data set out in Table 2 indicate, clustering scores increased linearly with category size for typical participants whilst for the ASD group clustering only increased notably with a category size of 12. This quadratic trend is significant (*F*(1, 37) = 5.36, *p* < .05). In contrast, an analysis of the IPC data revealed no significant main effects of group (*F*(1, 37) = 0.04, ns) or category size (*F*(1, 37) = 1.25, ns) and no interaction between these factors (*F*(4, 34) = 0.23, ns). Thus, our findings from the baseline condition confirm that without any support, participants with ASD use relational information to aid recall to a lesser extent than typical individuals, whereas their use of item-specific information to help their recall appears similar to that of the Comparison group.

### 3.2 Orienting tasks

Prior to analysing the recall performance following the orienting tasks, we assessed whether groups may have completed these tasks differently. During the category sorting task, participants in both groups performed at ceiling with only 3 ASD and 2 Comparison individuals committing either 1 or 2 errors. During the rating condition, ASD participants provided average ratings of 3.04 (S.D. = 0.41), which did not differ significantly from the average rating of 2.88 (S.D. = 0.27) given by the Comparison group. Similarly, an inspection of the frequency distributions of the ratings given by individuals revealed no differences between the groups. Taken together with the observation that groups did not differ significantly in terms of the time they spent looking through the deck of cards whilst they completed the orienting tasks these findings suggest no group differences in fulfilling the requirements of the orienting task instructions.

**Fig. 2:**
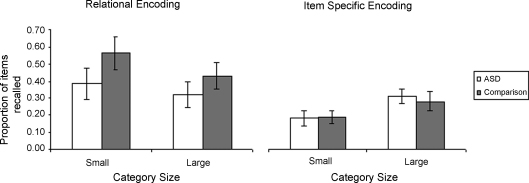
Average proportions of items recalled during the orienting task conditions from the small 2, 4 and 8 item categories and the large 12 and 16 item categories as a function of group and encoding condition. Error bars show standard errors.

Our analysis of the recall data following the orienting tasks (illustrated in Fig. 2) paralleled that of the baseline condition and encoding time was again entered as a covariate. A 2 (category size) by 2 (orienting task) by 2 (group) mixed ANCOVA of the recall data revealed a main effect of orienting task (*F*(1, 35) = 6.88, *p* < .05) indicating that recall following the relational encoding task (i.e. sorting words into categories) was superior to recall following the item-specific encoding task (i.e. rating words on pleasantness). The only other significant effect was an interaction between category size and orienting task (*F*(1, 35) = 12.79, *p* < .01), which replicates the findings reported by Hunt and Seta (1984). Post hoc comparisons showed that recall of items from the small categories was superior following the relational compared to the item-specific encoding task (*t*(38) = 4.15, *p* < .001; equal variance not assumed) whereas recall of items from the large categories was similar following either type of encoding task (*t*(38) = 1.51, *p* = .13). The lack of any interactions involving the group factor (*F*s < 1) and the absence of a main effect of group (*F*(1, 35) = 0.67, ns) suggests that the provision of support in the form of orienting tasks attenuated the free recall difficulties seen in ASD. One may criticise this latter conclusion on the grounds that the reduced group sizes during the two encoding conditions decreased the statistical power of the analysis of these data in comparison to the baseline condition. In relation to this issue three aspects of our data are worth further comment. Most important amongst these is the observation that unlike performance during the baseline condition, recall following the orienting task conditions was not characterised by interactions between group and category size for either the relational orienting task (*F*(1, 17) = 0.36, ns) or the item-specific orienting task (*F*(1, 17) = 0.13, ns). Thus the patterning of performance as a function of category size did no longer differ as a function of group. Second, Cohen's *d* effect sizes for the between group differences in recall of items from small categories were reduced from 0.96 during the baseline condition to 0.57 following the relational orienting task and 0.08 following the item-specific orienting task (respective effect sizes for larger categories were 0.44 and 0.21). Third, although order confounds and differences in encoding time (i.e. time spent looking through deck of word cards) make analyses across baseline and orienting task conditions problematic, inspection of the data set out in Figs. 1 and 2 show that performance of ASD individuals following the relational orienting task was nearly identical to the comparison groups’ performance during the baseline condition. Interestingly the item-specific orienting task reduced performance of comparison participants to the level of ASD individuals’ baseline performance. We will return to the implications of these results in more detail in our discussion.

Table 3 summarises the category availability, clustering and IPC data as a function of orienting task. As the category availability data suggest, overall recall of categories is generally better for larger categories (*F*(4, 32) = 24.97, *p* < .001) and following the relational orienting task (*F*(1, 35) = 12.04, *p* < .01). Furthermore, a significant interaction between category size and orienting task (*F*(4, 32) = 4.47, *p* < .01) indicates that the main effect of orienting task is mostly due to the increased availability of smaller categories following relational as compared to item-specific processing. Again the lack of a main effect of group or interactions involving the group factor (*F*s < 1) suggests that the effect of item-specific and relational orienting tasks on the recall of categories was similar for the two participant groups. An analysis of the clustering data revealed a main effect of category size (*F*(4, 32) = 8.54, *p* < .001) and a marginally significant orienting task by category size interaction (*F*(4, 32) = 2.58, *p* = .056), which follows Hunt and Seta's observation of larger differences in clustering between the item-specific and relational encoding conditions for the smaller as compared to the larger categories. Again the group factor did not yield a main effect (*F*(1, 35) = 1.39, *p* = .25) or interactions with the other factors (*F*s < 2). An analysis of the IPC data as a function of category size, group and orienting task, did not reveal any significant main effects or interactions (*F*s < 1.1), thus not replicating Hunt and Seta (1984) who reported higher IPC scores following the item-specific orienting task, especially for larger categories. In summary, these analyses are in line with the suggestion that recall performance in ASD is no longer characterised by disproportionate difficulties in drawing on relational information when orienting tasks constrain the processes by which information is encoded.

## 4 Discussion

In the current experiment we drew on a procedure developed by Hunt and Seta (1984) in order to evaluate the hypothesis that individuals with ASD are characterised by specific difficulties in relational memory processes. Furthermore we hoped to gain insights into the severity of such difficulties by assessing whether environmental support in the form of a relational orienting task could help individuals with ASD to employ such relational processes.

Our results from the baseline condition support previous demonstrations (e.g. Bowler et al., 1997; Smith et al., 2007; Tager-Flusberg, 1991) of reduced recall in individuals with ASD when categorical information is available to aid recall. The finding that the ASD group showed selectively reduced recall of smaller but not larger categories confirms our prediction that ASD is characterised by relatively specific difficulties in relational but not item-specific memory processes. Further support for this view stems from the finding that the ASD participants recalled overall fewer categories and were less likely than typical participants to cluster items into their respective categories during recall. In contrast, the ASD participants recalled as many items per category (IPC) as the Comparison group indicating that they make as much use of item-specific information to facilitate memory as typical individuals. Together these results strongly suggest that, in the absence of any support, individuals with ASD employ relational memory processes to facilitate recall to a lesser degree than typical individuals whilst their ability to draw on item-specific information to aid recall seems relatively intact.

Solely on the basis of the results from the baseline condition it is difficult to determine the severity of the relational memory difficulty evident in individuals with ASD. Our results from the supported encoding conditions shed some light on this issue. These results revealed that following item-specific and relational orienting tasks, overall recall performance between ASD and comparison participants were comparable. As noted in our results, we concede that this conclusion may be criticised on the basis of the reduced group sizes for each of the orienting task conditions, particularly because the ASD group's performance was numerically (if not significantly) worse than the typical group following the relational orienting task. What is crucial to note, however, is that unlike performance during the baseline condition the patterning of recall as a function of category size following the orienting tasks was very similar for the two groups as were the indices of relational and item-specific encoding. In other words, individuals with ASD no longer exhibited the disproportional difficulties with relational memory processes that characterised their performance during the baseline condition. In this context it is particularly noteworthy that the overall level of recall and the pattern of recall across category sizes of individuals with ASD following the relational orienting condition were almost identical to that of typical individuals during the baseline condition. Conversely, the comparison groups’ performance following the item-specific orienting task was nearly identical to that of the ASD group during the baseline condition. Thus, whilst the relational orienting task allowed individuals with ASD to achieve a level of performance comparable to that of typical individuals’ unsupported performance, the item-specific orienting task seems to have created a learning situation for typical individuals that mimics that experienced by individuals with ASD under normal circumstances.

A possible limitation of our observations from the orienting task conditions is the fact that all participants first completed the baseline condition. On the basis of this order confound it may be argued that individuals with ASD simply required more practice in order to employ relational memory processes successfully. Although problematic to some extent, our conclusions would not be altered even if the improvement in performance by individuals with ASD is to some extent attributable to disproportionate practice effects. In relation to the task support hypothesis (Bowler et al., 1997), the findings from the orienting task suggest that support in the form of an orienting task (and perhaps increased practice) helps individuals with ASD to overcome difficulties in deploying relational memory processes effectively. Thus our main conclusion is that rather than *lacking* the capacity to process relational information sufficiently to aid recall, individuals with ASD experience difficulties in *spontaneously* deploying them in a way that fosters effective learning and memory in novel and unsupported situations. This conclusion is in line with an argument developed by Mottron and colleagues (Mottron, 2004; Mottron, Dawson, Soulières, Hubert, & Burack, 2006) on apparent conceptual difficulties in ASD. Rather than accepting the view that higher-level conceptual processes are impaired in this population, these authors contend that enhanced low-level perceptual processes compete with higher-level integrative functions. In the domain of memory this competition may occur between item-specific and relational encoding processes.

As we have highlighted in our introduction, the distinction between item-specific and relational memory processes may not only prove useful in terms of understanding the behavioural pattern of memory functioning in individuals with ASD but it may also provide a fruitful heuristic framework for more direct investigations regarding the neuropathological correlates underlying memory functioning in this group. Since our observations are purely behavioural, we can only speculate about the neural underpinnings of the specific difficulties in relational memory processes that characterised performance of individuals with ASD in the current study. Given the evidence regarding morphological abnormalities of the hippocampus in ASD (e.g. Kemper & Bauman, 1998) and the growing evidence implicating this structure in relational memory processes, an appealing possibility is that the memory difficulties experienced by individuals with ASD stem from relatively specific functional atypicalities of hippocampally mediated memory processes (see also Nicolson et al., 2006). Although more direct neuroscientific investigations will be needed in order to specify the nature of this functional abnormality further, we would argue that at least two hypotheses may be generated on the basis of the current literature. Based on evidence suggesting that areas surrounding the hippocampus may under some circumstances mediate relational memory processes (see Eichenbaum, 2004 for a review), one possibility is that in ASD these adjacent areas are able to compensate for deficits in hippocampally mediated relational processes if environmental circumstances invite this level of processing. If environmental support is absent on the other hand, cortical areas adjacent to the hippocampus may simply perform their ‘default’ operations and mediate item-specific processes. Another possibility is that hippocampally mediated relational memory processes are principally intact but limited to such an extent that they are ineffectively deployed under spontaneous learning conditions. When environmental circumstances emphasise relational processes, however, this functional limitation may be sufficiently supported to permit a relatively typical behavioural expression of relational memory capacities. These two hypotheses are most likely not the only ones that may be put forward but we include them here to reinforce the point that the framework of item-specific versus relational memory processes provides a useful heuristic to generate future research to further specify the neural underpinnings of memory difficulties in ASD.

In summary, our observations provide strong support for the view that individuals with ASD exhibit relatively specific difficulties in the *spontaneous* deployment of relational memory processes. We stress the term *spontaneous* because we think it important to distinguish between an impairment in the ability to engage in otherwise normally functioning processes and processes that are so impaired that they cannot function normally under any circumstances. Our finding that individuals with ASD no longer exhibit disproportionately attenuated memory for smaller categories following a relational orienting task illustrates that supporting learning environments promote relational processes in this group. Future studies will be needed in order to determine whether the relational processes employed by individuals with ASD under supported conditions are mediated by the same hippocampal-based neural mechanisms as in typical individuals or whether adjacent brain areas which typically mediate item-specific memory processes compensate for atypical hippocampal functioning.

## Figures and Tables

**Table 1:** Age and IQ scores for the ASD and comparison groups as a function of orienting task

	ASD (*N* = 20)		Comparison (*N* = 20)	
	Sort	Rate	Sort	Rate
Age (years)				
*M*	34.3	33.9	30.4	36.8
S.D.	14.2	11.6	9.8	11.7

VIQ^a^				
*M*	102	100	104	102
S.D.	16	18	14	17

PIQ^b^				
*M*	101	95	103	104
S.D.	18	24	13	13

FIQ^c^				
*M*	102	97	104	103
S.D.	18	22	14	17

a Verbal IQ (WAIS-R^UK^ or WAIS-III^UK^).

b Performance IQ (WAIS-R^UK^ or WAIS-III^UK^).

c Full-scale IQ (WAIS-R^UK^ or WAIS-III^UK^).

**Table 2:** Means and standard deviations for indices of relational and item-specific encoding as a function of group and category size during the baseline condition

	Category size					
	2	4	8	12	16	Total^a^
ASD						
Categories recalled	.35 (.49)	.20 (.41)	.35 (.49)	.90 (.31)	1.0 (.00)	–
Items per category	.50 (.69)	.50 (1.24)	.34 (.69)	.31 (.27)	.28 (.25)	–
Clustering	.00 (.00)	.00 (.00)	.13 (.32)	.49 (.45)	.55 (.42)	.38 (.25)

Comparison						
Categories recalled	.35 (.49)	.80 (.41)	.90 (.37)	.90 (.31)	.90 (.31)	–
Items per category	.64 (.98)	.50 (.38)	.38 (.30)	.39 (.24)	.40 (.27)	–
Clustering	.10 (.31)	.28 (.45)	.48 (.44)	.66 (.40)	.52 (.39)	.46 (.26)

a This value does not represent the average across the different category sizes because all items are weighted equally towards this average.

**Table 3:** Means and standard deviations for indices of relational and item-specific encoding as a function of group, orienting tasks and category size

	Category size					
	2	4	8	12	16	Total^a^
Relational orienting task (sorting words into categories)						
ASD						
Categories recalled	.60 (.51)	.60 (.52)	.90 (.32)	.80 (.42)	1.0 (.00)	–
Items per category	.75 (.73)	.54 (.59)	.44 (.33)	.37 (.38)	.33 (.24)	–
Clustering	.20 (.42)	.30 (.49)	.61 (.50)	.46 (.50)	.57 (.41)	.45 (.26)
Comparison						
Categories recalled	.70 (.48)	.80 (.42)	.80 (.42)	.90 (.32)	.90 (.32)	–
Items per category	.86 (.66)	.66 (.43)	.72 (.42)	.54 (.35)	.44 (.30)	–
Clustering	.50 (.53)	.70 (.48)	.70 (.39)	.72 (.43)	.59 (.42)	.60 (.26)

Item-specific orienting task (rating words on pleasantness)						
ASD						
Categories recalled	.20 (.42)	.30 (.48)	.60 (.52)	.90 (.32)	1.0 (.00)	–
Items per category	1.0 (2.11)	.50 (.89)	.29 (.26)	.27 (.21)	.33 (.10)	–
Clustering	.20 (.42	.20 (.42)	.15 (.34)	.47 (.43)	.77 (.19)	.50 (.15)
Comparison						
Categories recalled	.10 (.32)	.20 (.42)	.80 (.42)	.80 (.42)	1.0 (.00)	–
Items per category	.50 (1.58)	.50 (1.05)	.31 (.22)	.31 (.33)	.27 (.15)	–
Clustering	.00 (.00)	.20 (.42)	.57 (.50)	.37 (.48)	.73 (.34)	.47 (.25)

a This value does not represent the average across the different category sizes because all items are weighted equally towards this average.

## Footnotes

1 Although the amygdala has also been extensively studied in ASD, this structure plays a specific role in memory for emotional stimuli (and other emotional processes) and will therefore not be considered in the current paper.

2 We thank James Hampton for his help in developing this criterion.

3 It is important to note that the CA and IPC measures are not entirely independent of the total number of words recalled. However, Burns and Brown (2000) have demonstrated that measures correcting for recall level ‘are inappropriate for comparisons in which one of the groups may not have used any relational information’ (p. 1062). Since we propose that individuals with ASD experience specific difficulties with relational memory processes it was therefore inappropriate to employ such corrections.
